# Efficacy and safety of BRAF inhibition alone versus combined BRAF and MEK inhibition in melanoma: a meta-analysis of randomized controlled trials

**DOI:** 10.18632/oncotarget.15632

**Published:** 2017-02-23

**Authors:** Mengdong Liu, Xuekang Yang, Jiaqi Liu, Bin Zhao, Weixia Cai, Yan Li, Dahai Hu

**Affiliations:** ^1^ Department of Burns and Cutaneous Surgery, Xijing Hospital, Fourth Military Medical University, Xi’an, China

**Keywords:** efficacy, adverse events, BRAF inhibition, MEK inhibition, melanoma

## Abstract

Recent clinical studies have shown that combination therapy of BRAF and MEK inhibition provides more survival benefit than BRAF inhibition monotherapy. However, the adverse events due to BRAF and MEK inhibitors impact the physical comfort and social life of patients. Thus, in this study we have undertaken a meta-analysis of randomized controlled trials to compare the efficacy and adverse events risk between monotherapy and combination therapy. We identified the relevant studies by searching PubMed, EMBASE and Google scholar databases, between the year January 2000 and May 2016. Based on the heterogeneity, the fixed- or random-effects models were employed to analyze the efficacy and the incidence rate of adverse events. In addition, the subgroup analyses were conducted to overcome the effects of heterogeneity. Finally, our study included five RCTs, involving 1730 patients for this meta-analysis. The fixed-effects model demonstrated that combination therapy of BRAF and MEK inhibition provided more survival benefit in terms of ORR, PFS and OS (P < 0.00001). But, the combination therapy also significantly increased the incidences of pyrexia, chills, vomiting, chorioretinopathy, retinal detachment, hypertension, night sweats, increased aspartate aminotransferase and creatine kinase levels (P < 0.05) as compared to monotherapy. But, based on the significantly better survival outcomes, the combined BRAF and MEK inhibition will obviously be the mainstay therapy for the BRAF V600-mutant melanoma. However, a set of adverse events should be paid attention when physicians consider combination therapy.

## INTRODUCTION

After the approval of BRAF inhibitor such as vemurafenib or dabrafenib by the United States Food and Drug Administration in 2011, there has been a significant improvement in the progression-free and overall survival in melanoma patients with metastasis and BRAF V600 mutation in comparison to chemotherapy [1, 2]. However, this monotherapy with BRAF inhibitors alone is restricted due to the development of acquired resistance in about half of the patients within 6–7 months of treatment [3–5]. The constitutive activation of mitogen-activated protein kinase (MAPK) pathway through mitogen-activated extracellular signal-regulated kinase (MEK) is considered as one of the main mechanisms of acquired resistance [6]. In recent years, several randomized clinical trials have shown that using a combination of BRAF inhibitor and mitogen-activated extracellular signal-regulated kinase (MEK) inhibitor, not only prevent or delay MAPK-driven acquired resistance but also improve the progression-free survival and overall survival [7, 8]. Despite these encouraging results, significant adverse events impacting the physical comfort and social life of patients, have been observed with the use of BRAF and MEK inhibitors and should not be overlooked [9]. Thus, we have conducted a meta-analysis of randomized controlled trials comparing the efficacy and risk of all the reported adverse events in melanoma between BRAF inhibition alone and combined BRAF and MEK inhibition.

## RESULTS

### Literature search

**Figure 1:**
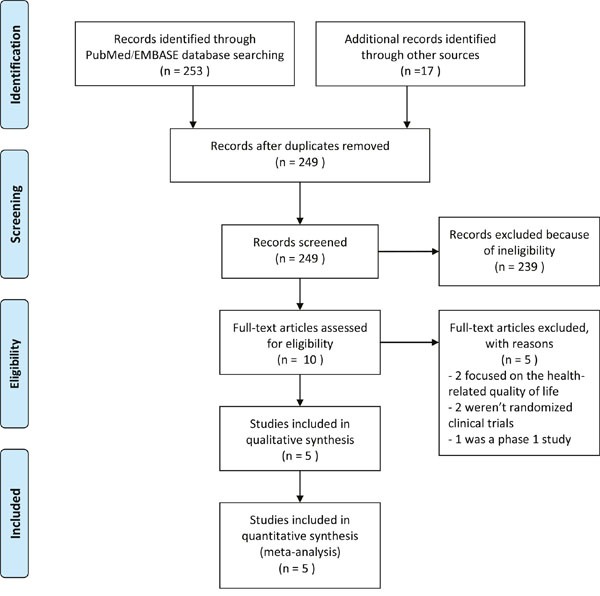
The schematic representation of study selection workflow

The complete study selection workflow has been schematically shown in Figure 1. A total of 270 citations were initially retrieved from PubMed, EMBASE and Google scholar databases and the majority of them were excluded based upon the assessment of abstracts or titles and because of them being reviews, case reports, animal trials, or irrelevant to our analytic aim. Among these 10 studies were considered potentially eligible. After full-text review of these 10 studies, 5 were excluded, because 2 of them mainly focused on the health-related quality of life [9, 10], 2 studies weren't randomized clinical trials [11, 12], and 1 study was only a phase 1 study [13]. Finally, 5 studies were eventually included in our meta-analysis [14–18]. Although data sources of patient information from 2 studies by Long *et al*. were same [14, 17], they were still included in our meta-analysis because they reported different kinds of adverse events. Moreover, when analyzed the same items from the 2 studies, we adopted the latest published data.

### Study characteristics and quality

**Figure 2:**
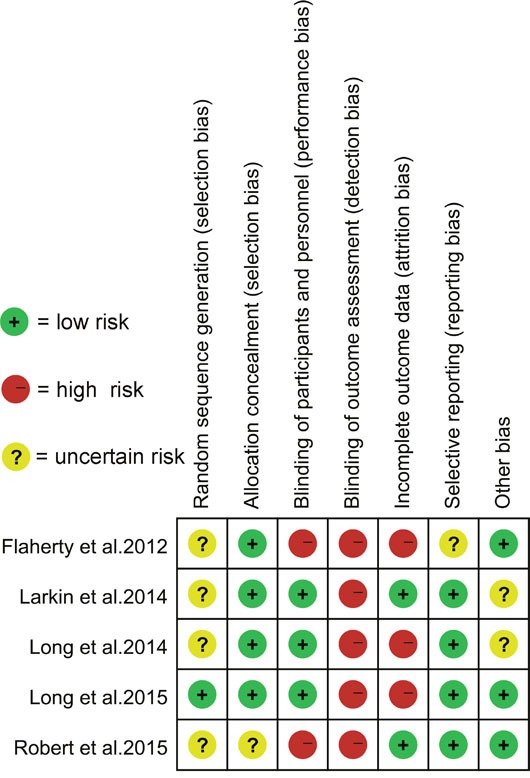
Risk of-bias assessment of randomized controlled trials included in meta-analysis

The main characteristics of the 5 included randomized clinical trials have been listed in Table 1. The size of the randomized clinical trials ranged from 162 to 704 (total 1784) patients. All studies except one used dabrafenib and trametinib as combination therapy. Only one study selected vemurafenib and cobimetinib as combination therapy. There were no significant differences in the baseline characteristics between combination therapy group and monotherapy group in any study. The studies by Robert *et al*. and Flaherty *et al*. were open-label studies, while a study by Long *et al*. was double-blind study. However, the study by Larkin *et al*. did not mention the blinding method. The quality assessment of the included studies has been listed in Figure 2.

**Table 1:** The main characteristics of RCTs included in the meta-analysis

Publication	Study type	Treatment regimen	Patients,n	Age,years*	Male sex,no. (%)	ORR	Median PFS,months	OS
Long et al.^16 #^2015	Phase III	Dabrafenib (150 mg, bid) and trametinib (2 mg, qd)	211	55 (22–89)	111 (53)	69%	11.0	74% at 12 months
	RCT	Dabrafenib (150 mg, bid) and placebo	212	57 (22–86)	114 (54)	53%	8.8	68% at 12 months
Robert et al.^17^2015	Phase III	Dabrafenib (150 mg, bid) and trametinib (2 mg, qd)	352	55 (18–91)	208 (59)	64%	11.4	72% at 12 months
	RCT	Vemurafenib only (960 mg, bid)	352	54 (18–88)	180 (51)	51%	7.3	65% at 12 months
Larkin et al.^18^2014	Phase III	Vemurafenib (960 mg, bid) and cobimetinib (60 mg, qd)	247	56 (23–88)	146 (59)	68%	9.9	81% at 9 months
	RCT	Vemurafenib (960 mg, bid) and placebo	248	55 (25–85)	140 (56)	45%	6.2	73% at 9 months
Long et al.^19 #^2014	Phase III	Dabrafenib (150 mg, bid) and trametinib (2 mg, qd)	211	55 (22–89)	111 (53)	67%	9.3	93% at 6 months
	RCT	Dabrafenib (150 mg, bid) and placebo	212	57 (22–86)	114 (54)	51%	8.8	85% at 6 months
Flaherty et al.^20^2012	Phase II	Dabrafenib (150 mg, bid) and trametinib (2 mg, qd)^&^	54	58 (27–79)	34 (63)	76%	9.4	79% at 12 months
	RCT	Dabrafenib (150 mg, bid) only	54	50 (18–82)	29 (54)	54%	5.8	70% at 12 months

*Data were expressed as median (range).

^&^Data of arm B (combination of dabrafenib 150 mg orally twice daily and trametinib 1 mg orally once daily) was not included in quantitative synthesis.

^#^Two trials by Long *et al*. were same and reported on two occasions, but included in our meta-analysis because they reported different kinds of adverse events. ORR = objective response rate, PFS = progression-free survival, OS = overall survival, RCT = randomized controlled trial, bid = twice a day, qd = once a day.

### Efficiency outcome

#### Overall response rate

**Figure 3:**
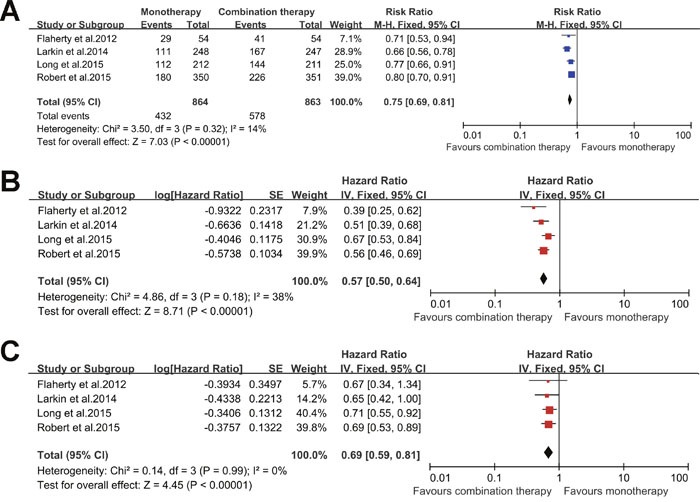
Forest plots analysis of the efficiency outcomes for combined BRAF and MEK inhibition versus BRAF inhibition alone **(A)** ORR; **(B)** PFS; and **(C)** OS.

All the included studied reported that the ORR of combination therapy was significant higher than that of monotherapy. Similarly, our fixed-effect model analysis also revealed that combination therapy significantly improved the ORR in comparison to monotherapy (RR: 0.75 [95% CI: 0.69 to 0.81], P < 0.00001; Heterogeneity: Chi^2^ = 3.50, df = 3 [P = 0.32], I^2^ = 14%) as shown in Figure 3A.

#### Progression-free survival

The information about HRs for PFS was also available from all trials. The pooled HR for PFS based on our fixed-effect model analysis demonstrated that combination therapy was associated with significantly longer PFS as compared to monotherapy (HR: 0.57 [95% CI: 0.50 to 0.64], P < 0.00001; Heterogeneity: Chi^2^ = 4.86, df = 3 [P = 0.18], I^2^ = 38%) as shown in Figure 3B.

#### Overall survival

Among the 5 studies, only 2 reported that combination therapy provided significant advantage in OS over monotherapy. Our fixed-effect model analysis also indicated that combination therapy was associated with a significant enhancement of OS (HR: 0.69 [95% CI: 0.59 to 0.81], P < 0.00001; Heterogeneity: Chi^2^ = 0.14, df = 3 [P = 0.99], I^2^ = 0%) as shown in Figure 3C.

### Adverse events

In our study, we analyzed the adverse events for all grades. A total of 44 different types of adverse events were recorded in the included studies. The fixed- or random-effects model analysis, based on the heterogeneity, revealed that combination therapy could significantly increased the incidence of pyrexia, chills, vomiting, chorioretinopathy, retinal detachment, hypertension, night sweats, increased aspartate aminotransferase and creatine kinase (P < 0.05). Meanwhile, the combination therapy also significantly decreased the incidence of arthralgia, hyperkeratosis, hand-foot syndrome, alopecia, skin papilloma and cutaneous squamous-cell carcinoma (P < 0.05). All the meta-analysis results about adverse events have been listed in Table 2.

**Table 2:** Outcomes of all-grade drug-related adverse events for combined BRAF and MEK inhibition versus BRAF inhibition alone

Adverse events	Trails	Risk-ration and 95%CI	P value	Heterogeneity	
				I^2^	P value
Pyrexia*	4	2.02 [1.40, 2.89]	0.0001	82%	0.0008
Chills	3	3.04 [1.88, 4.89]	< 0.00001	68%	0.04
Fatigue	3	1.06 [0.88, 1.27]	0.55	0%	0.37
Rash	4	0.85 [0.53, 1.35]	0.49	89%	< 0.00001
Nausea	4	1.39 [0.98, 1.98]	0.07	78%	0.004
Headache	2	1.11 [0.79, 1.56]	0.56	0%	0.89
Diarrhoea	4	1.45 [0.85, 2.48]	0.17	91%	< 0.00001
Arthralgia	4	0.67 [0.48, 0.93]	0.02	77%	0.005
Vomiting	4	1.85 [1.50, 2.29]	< 0.00001	0%	0.61
Aspartate aminotransferase increased	2	2.29 [1.13, 4.65]	0.02	56%	0.13
Oedema peripheral	2	2.95 [0.94, 9.27]	0.06	69%	0.07
Alanine aminotransferase increased	2	1.77 [0.83, 3.76]	0.14	66%	0.09
Dry skin	1	0.66 [0.38, 1.14]	0.14	/	/
Pruritus	1	0.66 [0.35, 1.23]	0.19	/	/
Hyperkeratosis	4	0.24 [0.16, 0.37]	< 0.00001	51%	0.11
Hand-foot syndrome	2	0.19 [0.13, 0.28]	< 0.00001	0%	0.37
Alopecia	4	0.22 [0.11, 0.45]	< 0.00001	84%	0.0003
Skin papilloma	3	0.09 [0.05, 0.16]	< 0.00001	3%	0.36
Dermatitis acneiform	2	1.54 [0.71, 3.36]	0.28	57%	0.13
Bleeding events	1	1.46 [0.64, 3.34]	0.37	/	/
Decrease in ejection fraction	4	4.12 [1.01, 16.81]	0.05	72%	0.01
Cutaneous squamous-cell carcinoma^&^	4	0.20 [0.10, 0.38]	< 0.00001	56%	0.08
Vision blurred	1	1.01 [0.26, 3.98]	0.99	/	/
Non-cutaneous malignancies	1	0.50 [0.09, 2.73]	0.43	/	/
Chorioretinopathy	4	9.52 [3.15, 28.76]	< 0.00001	48%	0.12
New primary melanoma	1	0.25 [0.03, 2.24]	0.22	/	/
Photosensitivity reaction	2	0.56 [0.05, 6.29]	0.64	98%	< 0.00001
Increased creatine kinase	1	10.22 [4.81, 21.71]	< 0.00001	/	/
Retinal detachment	1	40.47 [2.47, 664.40]	0.01	/	/
QT-interval prolongation	1	0.65 [0.28, 1.50]	0.31	/	/
Hypertension	2	1.66 [1.10, 2.50]	0.02	0%	0.60
Cough	2	1.09 [0.76, 1.57]	0.62	0%	0.33
Pain in a limb	1	0.92 [0.58, 1.45]	0.71	/	/
Decreased appetite	2	1.01 [0.65, 1.55]	0.98	0%	0.58
Abdominal pain	1	1.59 [0.83, 3.02]	0.16	/	/
Constipation	2	1.43 [0.87, 2.33]	0.16	0%	0.38
Myalgia	2	0.95 [0.62, 1.46]	0.83	0%	0.86
Asthenia	1	0.75 [0.43, 1.29]	0.30	/	/
Dizziness	1	1.68 [0.84, 3.35]	0.14	/	/
Nasopharyngitis	1	1.35 [0.71, 2.56]	0.36	/	/
Back pain	1	0.64 [0.37, 1.10]	0.11	/	/
Night sweats	1	4.33 [1.31, 14.35]	0.02	/	/
Elevated blood alkaline phosphatase	1	5.00 [0.60, 41.39]	0.14	/	/

*Pyrexia was defined as a body temperature of 38.5°C or higher. ^&^Keratoacanthoma was classified as cutaneous squamous-cell carcinoma.

### Subgroup analysis

**Figure 4:**
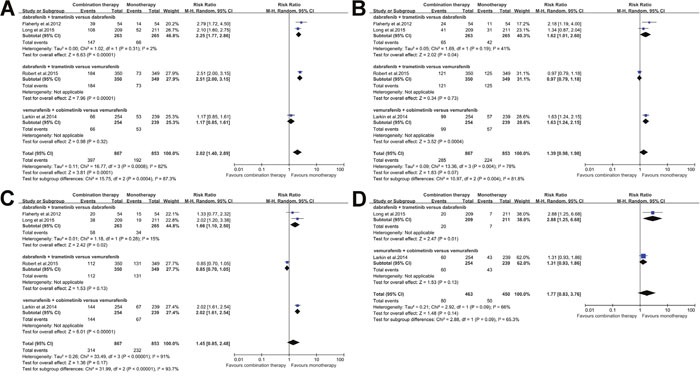
Subgroup analysis of the relative risk (RR) of all-grade adverse events for combined BRAF and MEK inhibition versus BRAF inhibition alone **(A)** Pyrexia; **(B)** Nausea; **(C)** Diarrhoea; and **(D)** increased Alanine aminotransferase.

**Figure 5:**
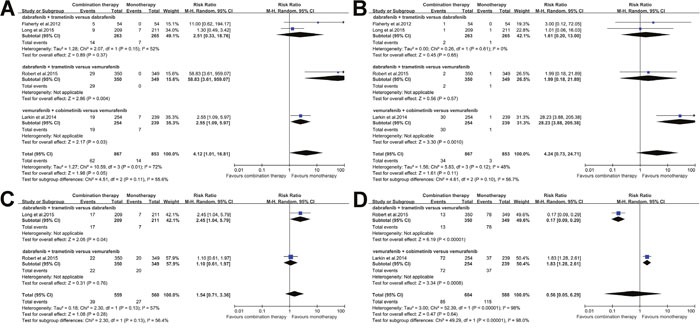
Subgroup analysis of the relative risk (RR) of all-grade adverse events for combined BRAF and MEK inhibition versus BRAF inhibition alone **(A)** Decrease in ejection faction; **(B)** Chorioretinopathy; **(C)** Dermatitis acneiform; and **(D)** Photosensitivity reaction.

As meta-analysis results about adverse events revealed heterogeneity, we further did the subgroup analysis according the treatment regimen. All the studies were classified into three subgroups: 1). dabrafenib + trametinib versus dabrafenib; 2) dabrafenib + trametinib versus vemurafenib; 3). vemurafenib + cobimetinib versus vemurafenib. The potential subgroup differences were observed in pyrexia, nausea, diarrhea, dermatitis acneiform, decrease in ejection fraction, chorioretinopathy and photosensitivity reaction, and increased alanine aminotransferase as illustrated in Figure 4 and 5. Further results of other subgroup analysis have been illustrated in Supplementary Figure 1 and 2.

### Publication bias

**Figure 6:**
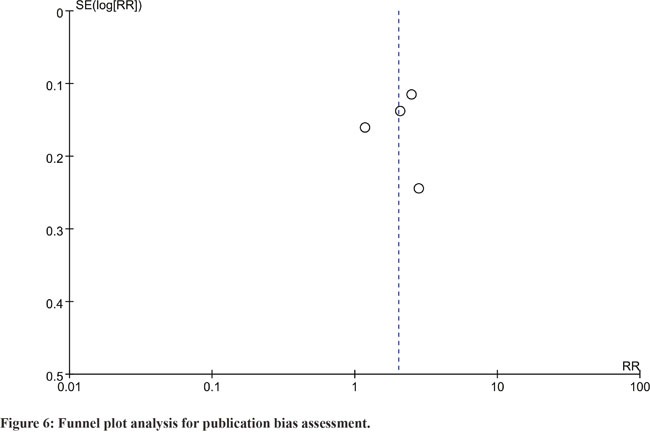
Funnel plot analysis for publication bias assessment

Publication bias was assessed using funnel plot produced by Review Manager 5.2 software. The funnel plot looked symmetrical, and thus indicated no significant publication bias (Figure 6).

## DISCUSSION

Approximately 40-50% of the cutaneous melanomas harbor oncogenic driver mutations at V600 codon in the serine-threonine kinase BRAF and this mutation induce constitutive activation of the MAPK signaling pathway [19–21]. The suppression of MAPK signaling by inhibiting BRAF has been observed to be an effective therapeutic strategy in BRAF V600-mutant melanomas. However, the onset of acquired resistance limits the efficacy of single-agent BRAF inhibitors. Several mechanisms mediating resistance to BRAF inhibitors through MAPK reactivation have been confirmed, including secondary NRAS or MEK mutations [22–24], amplification or alternate splicing of mutant BRAF [25, 26], CRAF upregulation [27], COT (MAP3K8) overexpression [28] and few other mechanisms [11]. These results advocated the use of combined therapy, by including downstream target inhibitor along with BRAF inhibitor, to prevent or delay the progression of acquired resistance. Recent clinical studies have shown that combined BRAF and MEK inhibition effectively forestalled the development of acquired resistance and achieved more survival benefit [14–18]. Consistent with this, the results of our meta-analysis also demonstrated that combined BRAF and MEK inhibition provided significant advantage in ORR, PFS and OS over BRAF inhibition alone [29]. Given the significantly better survival outcomes, the combined BRAF and MEK inhibition will become the mainstay therapy for BRAF V600-mutant melanoma. However, the reported success of these agents comes at the cost of a set of adverse events, which significantly impact the physical comfort and social life of patients [9]. Thus, in our study we conducted a comprehensive analysis of all these reported adverse events.

The secondary cancers like cutaneous squamous-cell carcinoma, skin papillomas and keratoacanthoma are considered to be the characteristic adverse events of BRAF inhibitor monotherapy, and occur in approximately 14 to 26% of the patients [30, 31]. The main reason for skin tumors development is the paradoxical activation of the MAPK pathway in keratinocytes with upstream activation of signaling by preexisting RAS mutations [32, 33]. Our meta-analysis demonstrated that the combination of BRAF and MEK inhibition, as compared with BRAF inhibition monotherapy, significantly decreased the incidence of secondary cancers. Beside these advantages, we also observed that combination of BRAF and MEK inhibition carried lower risks of arthralgia, hand-foot syndrome and alopecia than BRAF inhibition monotherapy.

Despite these advantages, the combination therapy of BRAF and MEK inhibition revealed some additional toxicities. Our meta-analysis demonstrated that combination therapy significantly increased the incidences of some adverse events, including pyrexia, chills, vomiting, increased aspartate aminotransferase, chorioretinopathy, increased creatine kinase, retinal detachment, hypertension and night sweats. Based on our further subgroup analysis, we also observed that combination of vemurafenib and cobimetinib therapy may carry higher risks of nausea, diarrhea, chorioretinopathy and photosensitivity reaction than the combination of dabrafenib and trametinib therapy. Moreover, subgroup analysis also showed that the risk of pyrexia due to combination of vemurafenib and cobimetinib therapy might be lower than the combination of dabrafenib and trametinib therapy. Based on the results of subgroup analysis, we believed that it was necessary for the physicians to adjust treat therapeutic regimens according to the severity and types of adverse events. However, further studies would be required to confirm these derived conclusions.

In addition, our study still has several limitations. First, the numbers of included studies were relatively small. More specifically, we only had 4 studies for analysis, as two studies had overlapping patient population. Thus limited numbers of patients for analysis can probability lead to reduced accuracy of our comparison results. Second, some adverse events were only reported in few studies, and thus again limiting the sample sizes for adverse events analysis. This limitation would further influence the reliability of our results. Finally, the treatment regimens were different among the included studies and the differences in drug and dose can also lead to certain errors while analyzing the adverse events. To address this concern, we conducted a series of subgroup analyses to overcome the effect of heterogeneity.

In conclusion, our current meta-analysis of five selected randomized controlled trials demonstrated that combination therapy of BRAF and MEK inhibition had significant survival benefit over BRAF inhibition monotherapy. In addition, our analysis displayed a specific set of adverse events which should be paid attention when using combination therapy. We hope that our results could provide a reference point for physicians in clinical practice when considering optimum treatment regimen for melanoma patients.

## MATERIALS AND METHODS

The PRISMA (Preferred Reporting Items for Systematic Reviews and Meta-analyses) guidelines were followed, while performing the present meta-analysis [34]. Since this study did not involve the access to direct patients or their samples, no ethical approval was required.

### Search strategy

To identify all potential studies related to the efficiency and adverse events of combined BRAF and MEK inhibition versus BRAF inhibition alone, a systematic literature search was conducted using PubMed, EMBASE and Google scholar databases between the year January 2000 to June 2016. The following search terms were used: “BRAF inhibition”, “MEK inhibition”, “dabrafenib”, “trametinib”, “vemurafenib”, “cobimetinib”, “melanoma”, “val600”, and “BRAF-mutant”. No restrictions were imposed and additionally reference lists of all the retrieved papers and recent reviews were further reviewed for identification of any relevant studies.

### Study selection

After the initial screening of titles/abstracts of the identified studies, secondary screening of the reviewed full-text was carried out. Studies were considered eligible if the following criteria were met: 1) The study design was a randomized clinical trial; 2) Enrolled patients had histologically confirmed metastatic melanoma with either BRAF V600E or BRAF V600K mutations and have received BRAF and MEK inhibition combination therapy or BRAF monotherapy; 3) efficiency measures included objective response rate (ORR), progression-free survival (PFS), overall survival (OS); and 4) adverse events were recorded.

### Data extraction and quality assessment

Data extraction was performed via standardized data-collection form, which included the following information: publication reference, treatment regimen, age, gender, ORR, PFS, OS and adverse events. Two investigators (M.D.L. and X.K.Y.) independently extracted the data and graded the methodological quality of each eligible study using the Cochrane Collaboration's risk-of-bias tool [35]. Any discrepancies were resolved by discussion with a third investigator (H.D.H.), or referencing the original publication.

### Statistical analysis

The risk ratio (RR) was used to summarize the results for ORR and all adverse events, while hazard ratio (HR) was used to summarize results for PFS and OS. The Mantel-Haenszel (MH) approach was implemented by either fixed- or random-effects models, based upon the heterogeneity in the included studies. The heterogeneities between the studies were assessed by I^2^ statistic, where values less than 50 percent represented no significant heterogeneity. In cases of no heterogeneity, the fixed effects model was used, and when heterogeneity was present, the random effects model was used and further subgroup analysis was conducted. A value of p<0.05 was considered statistically significant. Publication biases were assessed by visual examination of funnel plots. All analyses were performed via Review Manager (version 5.2, The Cochrane Collaboration, Oxford, UK) software.

## SUPPLEMENTARY MATERIALS FIGURES



## Abbreviations

MAPK: mitogen-activated protein kinase
MEK: mitogen-activated extracellular signal-regulated kinase
PRISMA: Preferred Reporting Items for Systematic Reviews and Meta-analyses
ORR: objective response rate
PFS: progression-free survival
OS: overall survival
RR: risk ratio
HR: hazard ratio
