# Supplementary material for: Efficacy and safety of BRAF inhibition alone versus combined BRAF and MEK inhibition in melanoma: a meta-analysis of randomized controlled trials
Source: Oncotarget. 2017 Feb 23;8(19):32258–69. doi: 10.18632/oncotarget.15632 (PMC5458282; doi:10.18632/oncotarget.15632)
Supplement: Supplementary file 1 [file oncotarget-08-32258-s001.pdf]

Efficacy and safety of BRAF inhibition alone versus combined BRAF and MEK inhibition in melanoma: a meta-analysis of randomized controlled trials

SUPPLEMENTARY FIGURES

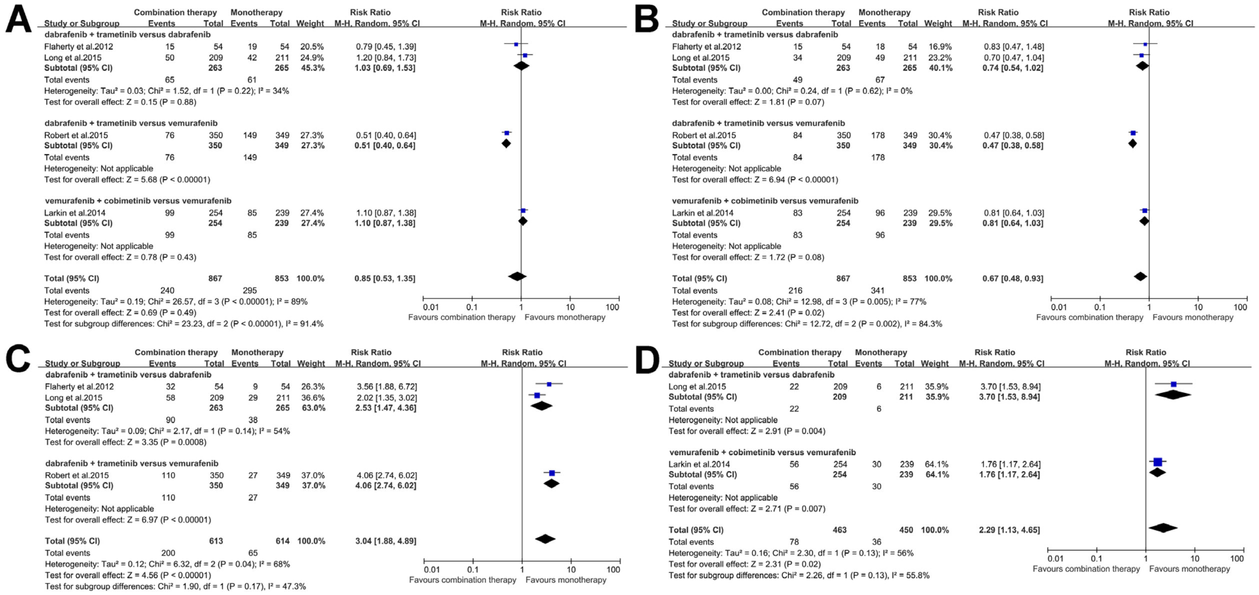

Supplementary Figure 1: Subgroup analysis of the relative risk (RR) of all-grade adverse events for combined BRAF and MEK inhibition versus BRAF inhibition alone. **(A)** Rash; **(B)** Arthralgia; **(C)** Chills; and **(D)** increased Aspartate Aminotransferase.

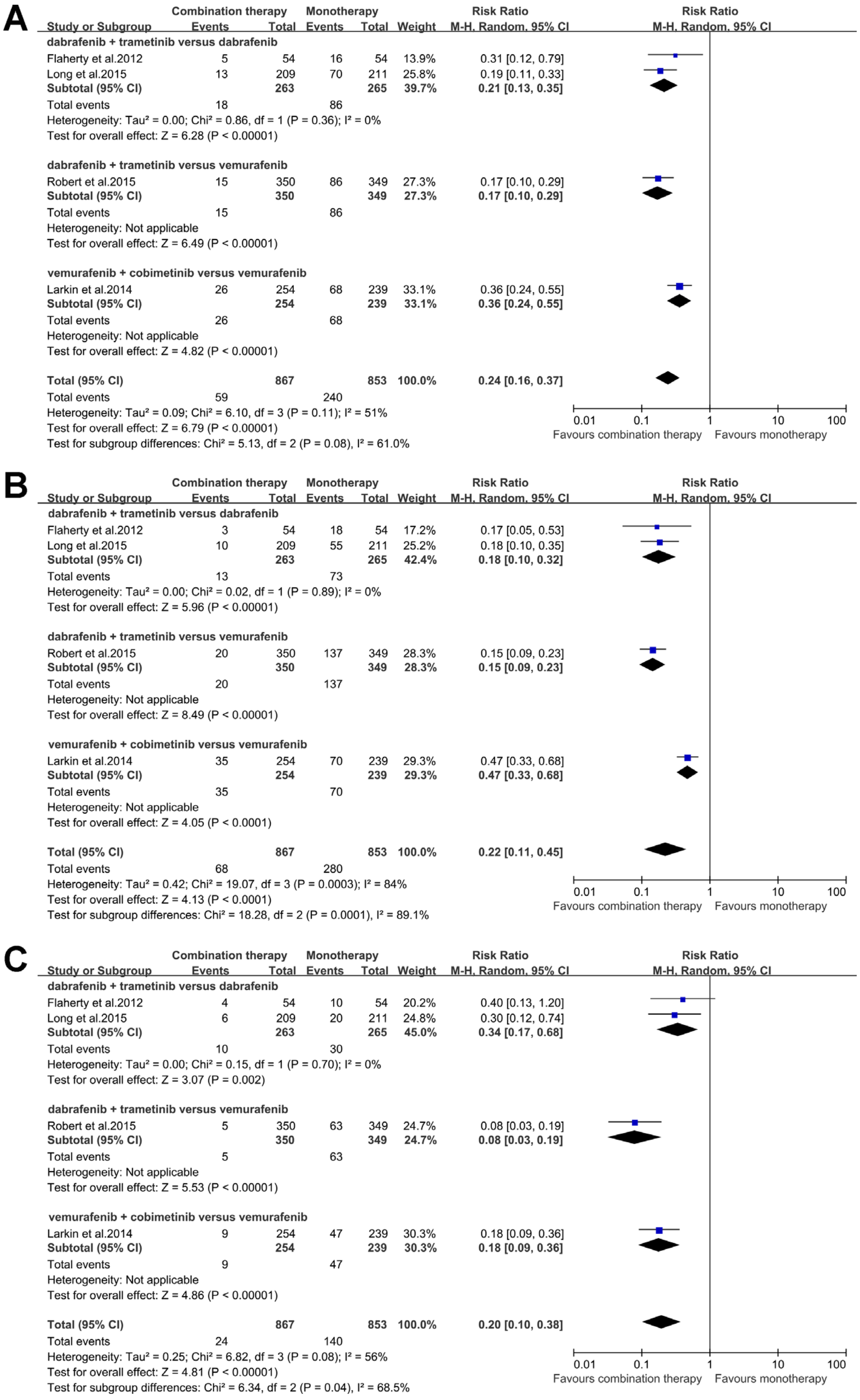

Supplementary Figure 2: Subgroup analysis of the relative risk (RR) of all-grade adverse events for combined BRAF and MEK inhibition versus BRAF inhibition alone. **(A)** Hyperkeratosis; **(B)** Alopecia; and **(C)** Cutaneous squamous-cell carcinoma.
